# The impact of preoperative malnutrition on postoperative delirium: a systematic review and meta-analysis

**DOI:** 10.1186/s13741-023-00345-9

**Published:** 2023-10-26

**Authors:** Bo Dong, Jing Wang, Pan Li, Jianli Li, Meinv Liu, Huanhuan Zhang

**Affiliations:** 1 Department of Anesthesiology, Hebei General Hospital, Shijiazhuang, 050051 China; 2 Graduate Faculty, Hebei North University, Zhangjiakou, 075132 China

**Keywords:** Malnutrition, Postoperative delirium, Meta-analysis, Systematic review

## Abstract

**Background:**

Postoperative delirium (POD) is a common postoperative complication, characterized by disturbance of attention, perception, and consciousness within 1 week after surgery, and linked to cognitive decline, increased mortality, and other serious surgical outcomes. Early identification and treatment of risk factors for POD could reduce the occurrence of delirium and the related poor outcomes. Malnutrition as a possible precipitating factor, defined as the poor anthropometric, functional, and clinical outcomes of nutrient deficiency, has been investigated. However, the evidence is controversial. The goal of this systematic review and meta-analysis was to comprehensively assess the correlation between preoperative malnutrition and POD.

**Methods:**

PubMed, Embase, Cochrane Library, and Web of Science were used to search prospective cohort articles that explored the correlation between preoperative malnutrition and POD from inception until September 30, 2022. Two researchers independently conducted the literature selection and data extraction. The quality of the literature was evaluated according to the Newcastle–Ottawa scale (NOS). Odds ratios (ORs) and 95% confidence intervals (CIs) for POD associated with malnutrition relative to normal nutritional status were calculated.

**Results:**

Seven prospective cohort studies qualified for the meta-analysis, which included 2701 patients. The pooled prevalence of preoperative malnutrition was 15.1% (408/2701), and POD occurred in 428 patients (15.8%). The NOS score was above 7 points in all 7 studies. Our results demonstrated that the pooled OR for malnutrition and POD was 2.32 (95% *CI* 1.62–3.32) based on a random-effects model. Our subgroup analysis suggested that the relationship between malnutrition and POD was significant in adults following noncardiac surgery (*OR* = 3.04, 95% *CI*, 1.99–4.62, *P* < 0.001), while there was no statistical significance in adults following cardiac surgery (*OR* = 1.76, 95% *CI*, 0.96–3.22, *P* = 0.07). Additionally, in the subgroup analysis based on different malnutrition assessment tools (MNA-SF versus others), a significant association was found in the MNA-SF group (*OR* = 3.04, 95% *CI*, 1.99–4.62, *P* < 0.001), but not in the others group (*OR* = 1.76, 95% *CI*, 0.96–3.22, *P* = 0.07). Other subgroup analyses showed that this association was not significantly affected by evaluation instruments for POD, location of the study, or quality of the article (all *P* < 0.05).

**Conclusions:**

Based on the currently available evidence, our results suggested that preoperative malnutrition was independently associated with POD in adult surgical patients.

**Supplementary Information:**

The online version contains supplementary material available at 10.1186/s13741-023-00345-9.

## Introduction

Postoperative delirium (POD), a relatively common and serious cerebral syndrome, is an acute and transient cerebral disorder characterized by disturbance of attention, perception, and consciousness within 1 week after surgery (Roy et al. 2019). The prevalence of POD varies from 11.1 to 45.6%, depending on the age of the patient and the type of surgery (Ho et al. 2021). POD can induce perioperative adverse events including prolonged hospital stay, increased medical expenses, lower probability of discharge to home, and some serious complications (Park and Kim 2019). In addition, it may also be associated with adverse long-term consequences including rehospitalizations and functional impairment, as well as an increased risk of dementia and mortality (Guenther et al. 2020; Mohanty et al. 2022). Therefore, appropriate prevention and treatment strategies for POD are essential for improving the prognosis and quality of life of patients. Unfortunately, the present pathophysiology of POD remains obscure, which hinders efforts to treat it (Jin et al. 2020). Based on the knowledge gaps in POD pathogenesis, the most effective management of POD is prevention (Jin et al. 2020). Our recent study reported some risk factors for POD including the age-adjusted Charlson Comorbidity Index (ACCI), preoperative MMSE scores, and others after thoracic and abdominal surgery (Liu et al. 2022). Moreover, the predisposing factors of malnutrition, frailty, and lower body mass index were associated with POD (Swarbrick and Partridge 2022). A previous study suggested that multicomponent risk intervention strategies were effective in preventing POD (Mossie et al. 2022). Thus, early preoperative identification and treatment of malnutrition might be an effective strategy for the prevention of POD.

Malnutrition is a condition characterized by changed body composition and body cell mass, resulting from starvation, comorbidities, or aging and leads to damaged physical and mental function and poor clinical outcomes (Bellanti et al. 2022). It is common in the elderly community setting, with an incidence from 0.8 to 24.6% (Crichton et al. 2019), while the prevalence is estimated to be 45% in elderly patients undergoing surgical procedures (Zhou et al. 2015). A growing body of studies has shown that malnutrition is associated with worse perioperative outcomes, including surgical site infections, readmission, prolonged hospital stay, and increased mortality (Hogan et al. 2022; Tóth et al. 2022; Tsantes et al. 2020). In addition, several studies have suggested that preoperative malnutrition might be related to the development of POD in surgical patients (Velayati et al. 2019; Mazzola et al. 2017). Moreover, the consensus-based guideline on POD of the European Society of Anesthesiology pointed out that malnutrition might be a predisposing factor for POD (Aldecoa et al. 2017). However, the relationship between preoperative malnutrition and POD has not been systematically reviewed. Understanding the nature of this association could enhance perioperative decisions to decrease the prevalence of POD and the related detrimental outcomes. Consequently, we thoroughly evaluated the association between preoperative malnutrition and POD by meta-analysis.

## Materials and methods

Our meta-analysis complied with the guidelines of the Preferred Reporting Items for Systematic Reviews and Meta-Analyses (PRISMA) (Liberati et al. 2009) and the Meta-Analyses and Systematic Reviews of Observational Studies in Epidemiology (MOOSE) (Stroup et al. 2000). The protocol was registered on the PROSPERO website (CRD42022373844).

### Literature search

PubMed, Cochrane Library, Web of Science, and Embase were searched from inception until September 30, 2022, using the following search keywords: (1) “malnutrition”OR”malnourishment”OR”nutritional status”OR”nutritional deficiency”OR”undernutrition”OR”nutrition disorder”; (2) “delirium”OR”deliri*”OR”postoperative delirium”OR”postoperative cognitive disorder”OR”confusion*”OR”mixed origin delirium”OR”subacute delirium”OR”acute confusional syndrome”OR”organic brain syndrome”OR”transient mental disorder”; and (3)”postoperative”OR”operation”OR”postoperative complications”OR”operati*”OR”surg*”OR”anesthes*”OR”anaesthes*”.

### Study selection

Prospective cohort studies were eligible for this review if they met all the following inclusion criteria: (1) adult surgical patients (> 18 years old); (2) preoperative malnutrition was identified using diagnostic criteria, nutritional screening tools, or serological laboratory values, including the Global Leadership Initiative on Malnutrition (GLIM) criteria, Nutrition Risk Screening-2002 (NRS-2002), Mini Nutritional Assessment (MNA), Mini Nutritional Assessment-Short Form (MNA-SF), Malnutrition Universal Screening Tool (MUST) or albumin; (3) POD can be assessed by diagnostic criteria or well-validated tools, such as the *Diagnostic and Statistical Manual of Mental Disorders 4th edition* (DSM-IV) or 5th edition (DSM-V), Confusion Assessment Method (CAM), Confusion Assessment Method Intensive Care Unit (CAM-ICU), or Delirium Observation Screening Scale (DOSS); (4) the study explored the relationship between preoperative malnutrition and POD; and (5) the study reported a crude or adjusted odds ratio (OR) of the relationship between preoperative malnutrition and POD or sufficient raw data to allow for calculation. The studies were excluded if they were as follows: (1) were review articles, letters, abstract-only publications, and case reports; (2) did not provide a specific definition of malnutrition or POD; and (3) included study populations with a history of neurological surgery, head trauma, psychiatric illness, or stroke.

### Data extraction and quality evaluation

Two researchers separately conducted the headline, abstract and full-text screening, data extraction, and quality evaluation of studies. Any disagreements were resolved through discussion. The extracted data included (1) study authors, (2) number of patients, (3) type of surgery, (4) measurement tools for malnutrition and its prevalence, (5) diagnostic methods for POD and its incidence, (6) crude OR, and (7) adjusted OR and adjusted variables. Quality assessment of included studies was rated using the Newcastle–Ottawa scale (NOS), which was recognized as a standardized method for quality assessment of nonrandomized studies (Stang 2010). The NOS mainly comprised three items: selection (4 items, 1 point each), comparability (2 items, 1 point each), and outcome (3 items, 1 point each), and the maximum score was 9 points. Studies with NOS scores ≥ 7.0 were considered high quality, and NOS scores < 7.0 were regarded as low quality.

### Statistical analysis

The relationship between preoperative malnutrition and POD was estimated by calculating odds ratios (ORs) and corresponding 95% confidence intervals (CIs). Adjusted OR was used in the meta-analysis when available. When the included studies did not report any ORs, unadjusted OR were calculated from raw data and combined with adjusted OR in others included studies to estimate the association of preoperative malnutrition with POD (Ceban et al. 2021). Forest plots were used to present the variation in OR estimates that expressed the correlation between preoperative malnutrition and POD. In view of the clinical diversity and high methodological heterogeneity between studies, we conducted the analysis using a random-effects model. We also performed subgroup analyses to evaluate the impacts of the location of study, type of surgery, quality of the study, and methods for evaluating malnutrition and POD on the correlation between preoperative malnutrition and POD. In addition, the credibility of statistically significant subgroup analysis was assessed using the Credibility of Effect Modification Analyses (ICEMAN) tool (Schandelmaier et al. 2020). Sensitivity analysis was conducted to evaluate the influence of each study on the stability of the available evidence. Funnel plots and Egger’s regression test were used to test publication bias. If there was publication bias, the trim-to-fill method was used to adjust it. Review Manager 5.3 and Stata 15.0 software were used to perform the data management and statistical analyses. *P* < 0.05 was considered statistically significant.

## Results

### Literature search

**Fig. 1:**
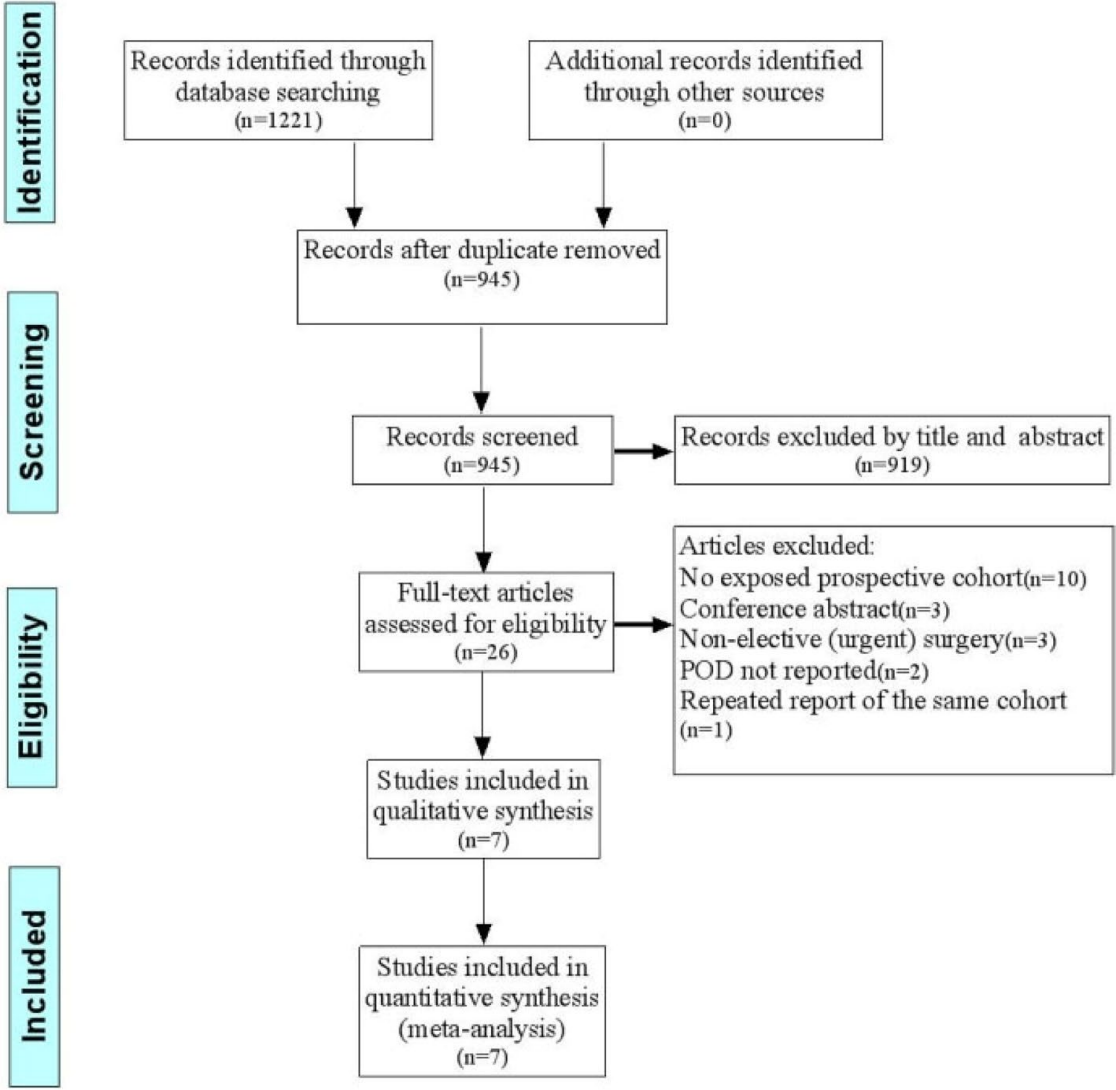
PRISMA flow diagram of study selection for the current meta-analysis

Figure 1 showed the detailed process of the database search. The initial literature search identified 1221 studies. After removal of duplicate articles by EndNote X9, we identified 945 articles. After preliminary headline and abstract screening, 919 citations were eliminated. The 26 remaining studies consequently were subjected to full-text review. Subsequently, 19 studies were removed for the following reasons: no exposed prospective cohort (10 studies), conference abstract (3 studies), nonelective (urgent) surgery (3 studies), POD not reported (2 studies), and repeated report of the same cohort (1 study). Ultimately, 7 articles were eligible for this meta-analysis.

### Description of included studies

Table 1 presented the main characteristics of the selected studies. In total, 7 prospective cohort studies published from 2015 to 2020 involved 2701 patients (average age of 74 years), of whom 51% were male. Of the 7 studies, 3 studies were from Asia (Velayati et al. 2019; Chu et al. 2016; Zhao et al. 2020), and 4 were from Europe (Mazzola et al. 2017; Ringaitiene et al. 2015; Wulp et al. 2021; Sánchez Acedo et al. 2020). Three studies included cardiac surgical patients (Velayati et al. 2019; Ringaitiene et al. 2015; Wulp et al. 2021), and the other 4 studies involved noncardiac surgical patients (Mazzola et al. 2017; Chu et al. 2016; Zhao et al. 2020; Sánchez Acedo et al. 2020). Malnutrition was diagnosed using the MNA-SF in 4 studies (Mazzola et al. 2017; Chu et al. 2016; Zhao et al. 2020; Sánchez Acedo et al. 2020), NRS-2002 in 2 studies (Velayati et al. 2019; Ringaitiene et al. 2015), and MNA or albumin in 1 study (Wulp et al. 2021). Overall, the incidence of malnutrition varied from 5 to 26% across studies with a pooled incidence of 15.1%. Four studies adopted the CAM to assess delirium (Velayati et al. 2019; Zhao et al. 2020; Ringaitiene et al. 2015; Sánchez Acedo et al. 2020), in which 2 used the CAM-ICU (Velayati et al. 2019; Ringaitiene et al. 2015), and 3 others used the DSM-IV (Mazzola et al. 2017; Chu et al. 2016; Wulp et al. 2021). The pooled incidence of POD was 15.8% (range from 8 to 29.9%). Of the 7 studies in this review, 6 articles provided adjusted ORs for potential confounding factors including age, comorbidities, sex, surgery type, and ASA score (Velayati et al. 2019; Mazzola et al. 2017; Chu et al. 2016; Zhao et al. 2020; Wulp et al. 2021).

**Table 1:** Characteristics of the included studies

Study (year)	Patients (*n*)	Type of surgery	Definitions of malnutrition	Percent malnutrition	Assessment of POD	Percent POD	Crude OR (95% *CI*)	Adjusted OR (95% *CI*)	Multivariable adjustment
Ringaitienė et al. (2015)	99	CABG	NRS-2002 > 3	24.0	CAM-ICU	8.0	6.316 (1.384–28.819)	6.316 (1.384–28.819)	STS predicted mortality/morbidity
Chu et al. (2016)	544	Orthopedic surgery	MNA-SF < 12	17.5	DSM-IV	9.6	4.63 (2.54–8.44)^a^	2.85 (1.19–6.87)	Age, male gender, living status, admission route, BMI, IADL, sodium level, Barthel index, CCI, MMSE, GDS-15, type of surgery, time from admission to surgery, blood transfusion during operation, hearing impairment
Mazzola et al. (2017)	415	Hip fracture	MNA-SF ≤ 7	18.8	DSM-IV	29.9	6.3 (3.3–12.2)	3.0 (1.4–6.2)	Age, sex, CCI, ADL, ASA, preoperative cognitive status
Velayati et al. (2019)	398	CABG	NRS-2002 > 3	5.0	CAM-ICU	17.0	1.76 (1.02–3.05)	1.56 (1.20–3.24)	Age, hyperlipidemia, hypertension, vision impairment, creatinine, and sodium level after surgery
van der Wulp et al. (2021)	511	TAVI	Albumin < 3.5 or MNA < 12	26.0	DSM-IV	12.9	1.8 (1.0–3.2)	1.3 (0.6–2.7)	Age, BMI, previous delirium, aortic valve area < 0.75
Sánchez Acedo et al. (2020)	446	Urgent abdominal surgery	MNA-SF ≤ 7	7.0	CAM	13.6	2.54 (1.07–6.00)a	Not reported	Not reported
Zhao et al. (2020)	288	Noncardiac surgery	MNA-SF ≤ 7	14.2	CAM	17.0	6.70 (2.87–15.62)^a^	4.06 1.62–10.18)	Age, sex, CCI, depression, Barthel index, preoperative pain

*Abbreviations*: *BMI* body mass index, *CI* confidence interval, *CABG* coronary artery bypass graft, *TIVA* transcatheter aortic valve implantation, *CCI* Charlson Comorbidity Index, *POD* postoperative delirium, *CAM* Confusion Assessment Method, *CAM-ICU* Confusion Assessment Method for the Intensive Care Unit, *DSM-IV Diagnostic and Statistical Manual of Mental Disorders, Fourth Edition*, *IADL* instrumental activities of daily living, *GDS-15* 15-item Geriatric Depression Scale, *ADL* activities of daily living, *STS* Society of Thoracic Surgeons, *ASA* American Society of Anesthesiologists, *MMSE* Mini-Mental-State Examination, *MNA-SF* Mini Nutrition Assessment-Short Form, *MNA* Mini Nutrition Assessment, *NRS-2002* nutritional risk screening 2002

^a^Based on our own calculation, abstracted from raw data

### Quality evaluation

The quality of the selected studies was assessed according to NOS, and the NOS score varied from 7 to 9 in all studies, suggesting a high quality of all articles (Table 2).

**Table 2:** The Newcastle–Ottawa scale (NOS) scoring

Study (year)	Selection				Comparability		Outcome			Total
	Representativeness of the exposed cohort	Selection of the nonexposed cohort	Ascertainment of exposure	Outcome not present at baseline	Control for age	Control for other confounding factors	Assessment of outcome	Enough long follow-up duration	Adequacy of follow-up of cohorts	
Ringaitienė et al. (2015)	1	1	1	1	0	1	1	1	1	8
Chu et al. (2016)	1	1	1	1	1	1	1	1	1	9
Mazzola et al. (2017)	1	1	1	1	1	1	1	1	1	9
Velayati et al. (2019)	1	1	1	1	1	1	1	1	1	9
van der Wulp et al. (2021)	1	1	1	1	1	1	1	1	1	9
Sánchez Acedo et al. (2020)	1	1	1	1	0	0	1	1	1	7
Zhao et al. (2020)	1	1	1	0	1	1	1	1	1	8

### Preoperative malnutrition and POD

**Fig. 2:**
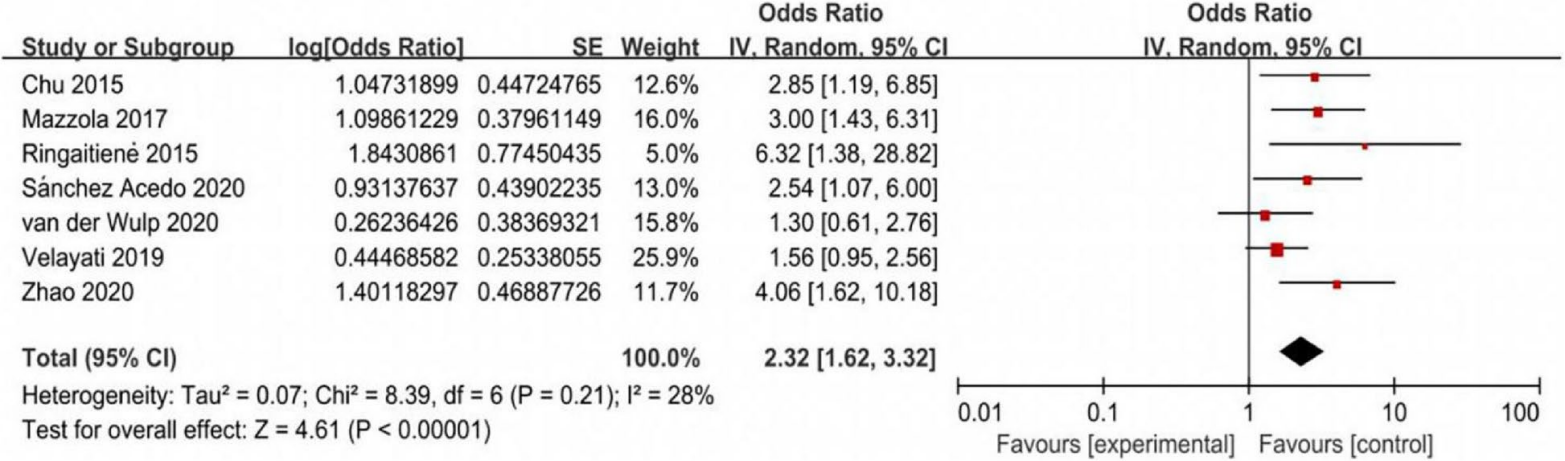
Forest plot for adjusted association between preoperative malnutrition and postoperative delirium

Based on a random-effects model, the pooled analysis of the 7 prospective cohort articles indicated a statistically significant relationship between preoperative malnutrition and POD (*OR* = 2.32, 95% *CI*, 1.62–3.32, *P* < 0.001; Fig. 2).

### Subgroup analysis results

Our subgroup analysis suggested that the relationship between malnutrition and POD was significant in adults following noncardiac surgery (*OR* = 3.04, 95% *CI*, 1.99–4.62, *P* < 0.001), while there was no statistical significance in adults following cardiac surgery (*OR* = 1.76, 95% *CI*, 0.96–3.22, *P* = 0.07). Additionally, in the subgroup analysis based on different malnutrition assessment tools (MNA-SF versus others), a significant association was found in the MNA-SF group (*OR* = 3.04, 95% *CI*, 1.99–4.62, *P* < 0.001), but not in the others group (*OR* = 1.76, 95% *CI*, 0.96–3.22, *P* = 0.07). Other subgroup analyses showed that this association was not significantly affected by evaluation instruments for POD, location of the study, or quality of the article (all *P* < 0.05; Table 3). The results of the subgroup analysis were also presented as forest plots in Additional file 1: Figure S1–S5. In addition, there was no significant interaction between any subgroup (all *P* for interaction > 0.05; Table 3). Thus, the use of ICEMAN criteria to assess the subgroup effects was not applicable in our meta-analysis.

**Table 3:** Subgroup analysis by study characteristics on the association between preoperative malnutrition and postoperative delirium

Study characteristics	Association between malnutrition and POD				
	Studies number	OR (95% *CI*)	*I*^2^	*P* for subgroup effect	*P* for interaction
Location					0.97
Asian	3	2.36 (1.30 − 4.28)	48%	< 0.01	
European	4	2.40 (1.39 − 4.13)	33%	< 0.01	
Type of surgery					0.15
Cardiac	3	1.76 (0.96 − 3.22)	42%	0.07	
Non-cardiac	4	3.04 (1.99 − 4.62)	0%	< 0.001	
Malnutrition evaluation					0.15
MNA-SF	4	3.04 (1.99 − 4.62)	0%	< 0.001	
Others	3	1.76 (0.96 − 3.22)	42%	0.07	
POD diagnosis					0.68
CAM	4	2.59 (1.45 − 4.63)	46%	< 0.01	
DSM-IV	3	2.20 (1.27 − 3.79)	31%	< 0.01	
Study quality					0.09
NOS 7–8	3	3.50 (1.96 − 6.25)	0%	< 0.001	
NOS 9	4	1.92 (1.30 − 2.83)	22%	< 0.01	

*Abbreviations*: *OR* odds ratio, *CI* confidence interval, *MNA-SF* Mini Nutrition Assessment-Short Form, *CAM* Confusion Assessment Method, *DSM-IV Diagnostic and Statistical Manual of Mental Disorders, Fourth Edition*, *NOS* the Newcastle–Ottawa scale

### Publication bias and sensitivity analysis

**Fig. 3:**
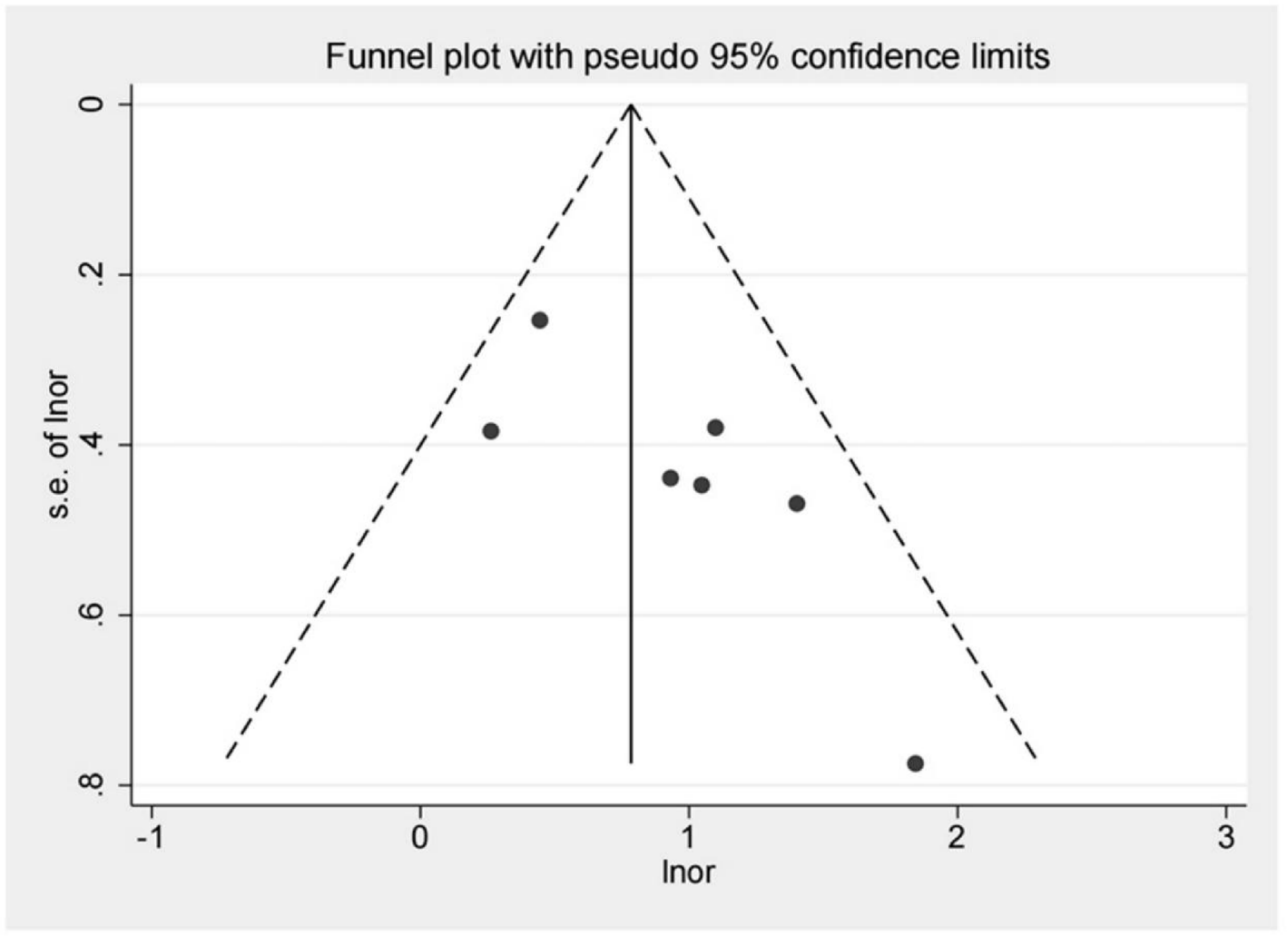
Publication bias was assessed through the visual examination of a funnel plot

**Fig. 4:**
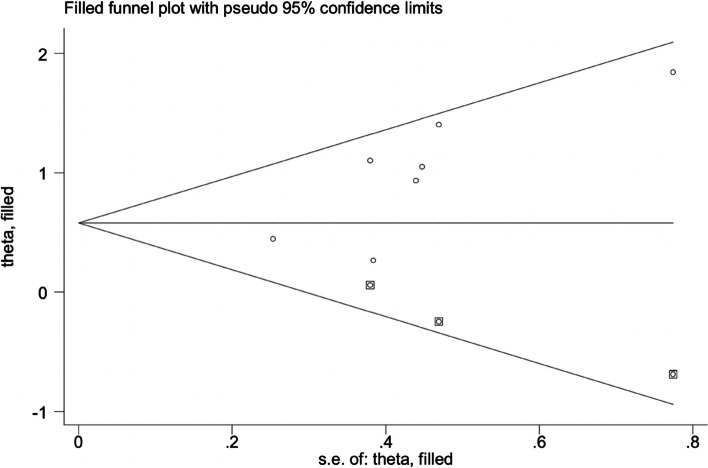
Publication bias was adjusted through the trim-to-fill method

**Fig. 5:**
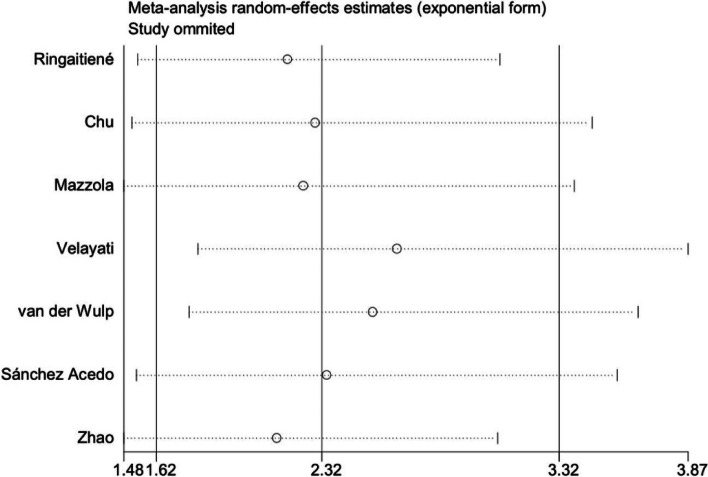
Sensitivity analysis for each study on the summary effect

The funnel plot was asymmetric (Fig. 3), and Egger’s regression test was significant (*P* = 0.037), indicating that significant publication bias was found in our study. Next, we further adjusted the publication bias using a trim-to-fill analysis, and the results revealed that 3 articles were missing (Fig. 4). After adding the potential missing 3 studies, the combined results did not change significantly (*OR* = 1.83, 95% *CI*, 1.25–2.68, *P* < 0.001), suggesting that the outcome of our meta-analysis was reliable. In addition, the results of the sensitivity analysis were reliable and robust with an OR from 2.13 (95% *CI*, 1.48–3.06) to 2.64 (95% *CI*,1.80–3.87), indicating that no study could change the results (Fig. 5).

## Discussion

To the best of our knowledge, this was the first meta-analysis to evaluate the correlation between preoperative malnutrition and POD in adult surgical patients. We identified 7 prospective cohort studies enrolling 2408 patients, and our results showed that malnutrition occurred in 22% of adult surgical patients, and preoperative malnutrition was a significant predictor of POD after surgery. Hence, studies exploring the effect of early identification and treatment of malnutrition on the incidence of POD should be considered in the future.

The specific mechanisms underlying the association between preoperative malnutrition and POD have not been elucidated. In fact, nutrition plays a critical role in all functions of the body, particularly brain function. In addition, previous studies have demonstrated that malnutrition is significantly associated with cognitive impairment and functional impairment (Sun et al. 2021; Amasene et al. 2021), all of which are risk factors for POD. Recent research has also showed that lack of a certain nutrients plays an important role in the progression of delirium (Sanford and Flaherty 2014). In this study, our meta-analysis results demonstrated that preoperative malnutrition could increase the risk of POD. However, it is important to note that included studies considered several factors that may confound the association between preoperative malnutrition and POD, but we could not exclude potential confounding factors. Patients with malnutrition were usually accompanied with systemic inflammation response, while inflammation played a critical role in the pathophysiology of POD (Pang et al. 2022). The association of malnutrition with POD might be attributed to the systemic inflammation response. Understanding the role of inflammation in the association between malnutrition and POD might provide new ideas and strategies to diminish the impact of malnutrition on POD. Moreover, frailty and sarcopenia were important confounding factors for the association between preoperative malnutrition and POD. Malnutrition, sarcopenia, and frailty are common diseases in hospitalized patients (Ligthart-Melis et al. 2020). A previous study showed that malnutrition played a major role in the development of frailty and sarcopenia (Cruz-Jentoft et al. 2017). Notably, frailty and sarcopenia have been identified as risk factors for POD (Gracie et al. 2021; Makiguchi et al. 2020). Thus, it is important to investigate the effect of sarcopenia or frailty on the correlation between malnutrition and POD in future studies.

Given the adverse prognosis of POD, researchers have focused on the management of POD to decrease its incidence and the related detrimental outcomes. Currently, early identification of risk factors and implementation of targeted interventions are regarded as effective strategies for preventing POD (Hughes et al. 2020). Based on the results of our meta-analysis, we hypothesized that early identification and treatment of malnutrition might decrease the prevalence of POD. Indeed, a randomized controlled trial showed that nutrition intervention might lead to a shorter duration of POD in the hip fracture patients (Olofsson et al. 2007). Although another randomized study demonstrated that multifactorial interventions, including nutrition intervention, could decrease the occurrence and duration of POD after surgery (Unal et al. 2022), whether nutrition intervention alone could effectively decrease the occurrence of POD remained unknown. In addition, there were multiple nutritional interventions (e.g., oral nutritional supplements, food modification, dietary advice) in the prevention or treatment of malnutrition (Dent et al. 2023). Therefore, it was imperative to choose the appropriate nutritional interventions to eliminate the negative impact on POD in adult surgical patients.

The prevalence of POD varies widely depending on the type of surgery, assessment method, and frequency of assessment (Hughes et al. 2020). It has been reported that the incidence of POD after cardiac surgery is higher than that after noncardiac surgery due to the disease itself and the severity of surgical trauma (Watt et al. 2018). Patients undergoing cardiac operation usually have a high ASA level, which is related to more comorbidities (diabetes, stroke, depression, chronic pain, dementia, cardiovascular and peripheral vascular diseases). These comorbidities are also risk factors for POD (Liu et al. 2022). In addition, cardiac surgery requires aortic cross-clamp, cardiopulmonary bypass, and longer operation and anesthesia time, all of which increase the incidence of POD (Rengel et al. 2023). Our subgroup results suggested that the association between malnutrition and POD was significant in adults following noncardiac surgery, while there was no statistical significance in adults following cardiac surgery, which could be explained by patients undergoing cardiac surgery and having many other risk factors for developing POD. The results highlighted the importance of assessing malnutrition before noncardiac surgery.

Studies using different malnutrition or POD assessment methods were obtained in this meta-analysis. Several malnutrition screening and assessment methods were adopted to identify malnutrition patients. However, there is no gold standard to define malnutrition in clinical and research settings. In our study, four studies measured malnutrition using the MNA-SF, NRS-2002 in 2 studies, and MNA or albumin in 1 study. However, our subgroup analysis suggested that a significant association of malnutrition with POD was found in the MNA-SF group, but not in the other group. Thus, it would be reasonable to assume that MNA-SF might be an appropriate malnutrition assessment tool for predicting the occurrence of POD. Similarly, there was a wide range of POD screening tools in our meta-analysis. DSM-IV, CAM, and CAM-ICU were used for POD diagnosis. Despite the variability in the methods used to assess POD, we found consistent results in the subgroup analysis assessing the association between malnutrition and POD. In addition, this association was not significantly affected by location of the study, or quality of the article, indicating that the outcome of our meta-analysis was reliable.

Several limitations of the present study should be considered. First, studies with various POD and malnutrition assessment tools were included in the meta-analysis, which limited the generalizability of our results. Second, a small number of literatures were included for this review, which hindered further meta-regression analyses. Third, funnel plots showed possible publication bias in current data, which might weaken the validity of our conclusions. Fourth, due to the study from Sanchez Acedo et al. who did not report adjusted OR (Sánchez Acedo et al. 2020), we used unadjusted OR in our meta-analysis, which might result in some degree of bias. Finally, the causative association between preoperative malnutrition and POD could not be concluded due to limited availability of information. Accordingly, future multicenter studies should be conducted to further elucidate the correlation between preoperative malnutrition and POD.

## Conclusion

Taken together, we found evidence of a significant relationship between preoperative malnutrition and POD in our meta-analysis. Future studies are warranted to evaluate the accuracy and feasibility of various preoperative malnutrition assessment tools to identify surgical patients at higher risk for POD.

### Supplementary Information

**Additional file 1: Supplementary Fig. S1.** Forest plot for the subgroup analysis based on location of study. **Supplementary Fig. S2.** Forest plot for the subgroup analysis based on type of surgery. **Supplementary Fig. S3.** Forest plot for the subgroup analysis based on methods for evaluating malnutrition. **Supplementary Fig. S4.** Forest plot for the subgroup analysis based on methods for evaluating POD. **Supplementary Fig. S5.** Forest plot for the subgroup analysis based on quality of the study.

## Data Availability

The datasets used and/or analyzed during the current study are available from the corresponding author on reasonable request.

## Abbreviations

POD: Postoperative delirium
NOS: Newcastle-Ottawa scale
OR: Odds ratio
CIs: Confidence intervals
ACCI: Age-adjusted Charlson Comorbidity Index
PRISMA: Preferred Reporting Items for Systematic Reviews and Meta-Analyses
MNA-SF: Mini Nutrition Assessment-Short Form
NRS-2002: Nutritional risk screening 2002
MNA: Mini Nutrition Assessment
CAM: Confusion Assessment Method
CAM-ICU: Confusion Assessment Method for the Intensive Care Unit
DSM-IV: Diagnostic and Statistical Manual of Mental Disorders
