# Supplementary material for: The impact of preoperative malnutrition on postoperative delirium: a systematic review and meta-analysis
Source: Perioper Med (Lond). 2023 Oct 26;12:55. doi: 10.1186/s13741-023-00345-9 (PMC10604920; doi:10.1186/s13741-023-00345-9)

**Additional file 1**

**Supplementary Figure S1.** Forest plot for the subgroup analysis based on location of study.


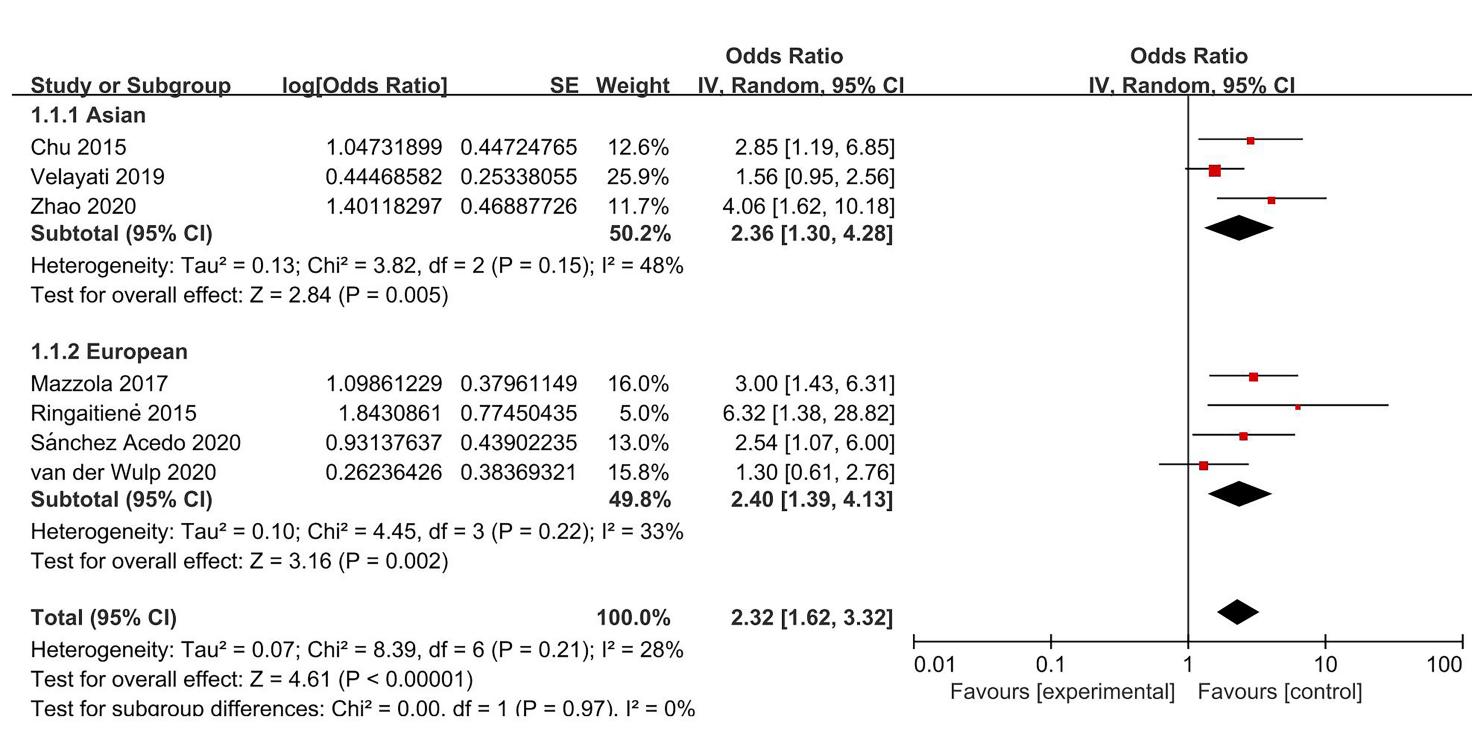


**Supplementary Figure S2.** Forest plot for the subgroup analysis based on type of surgery.


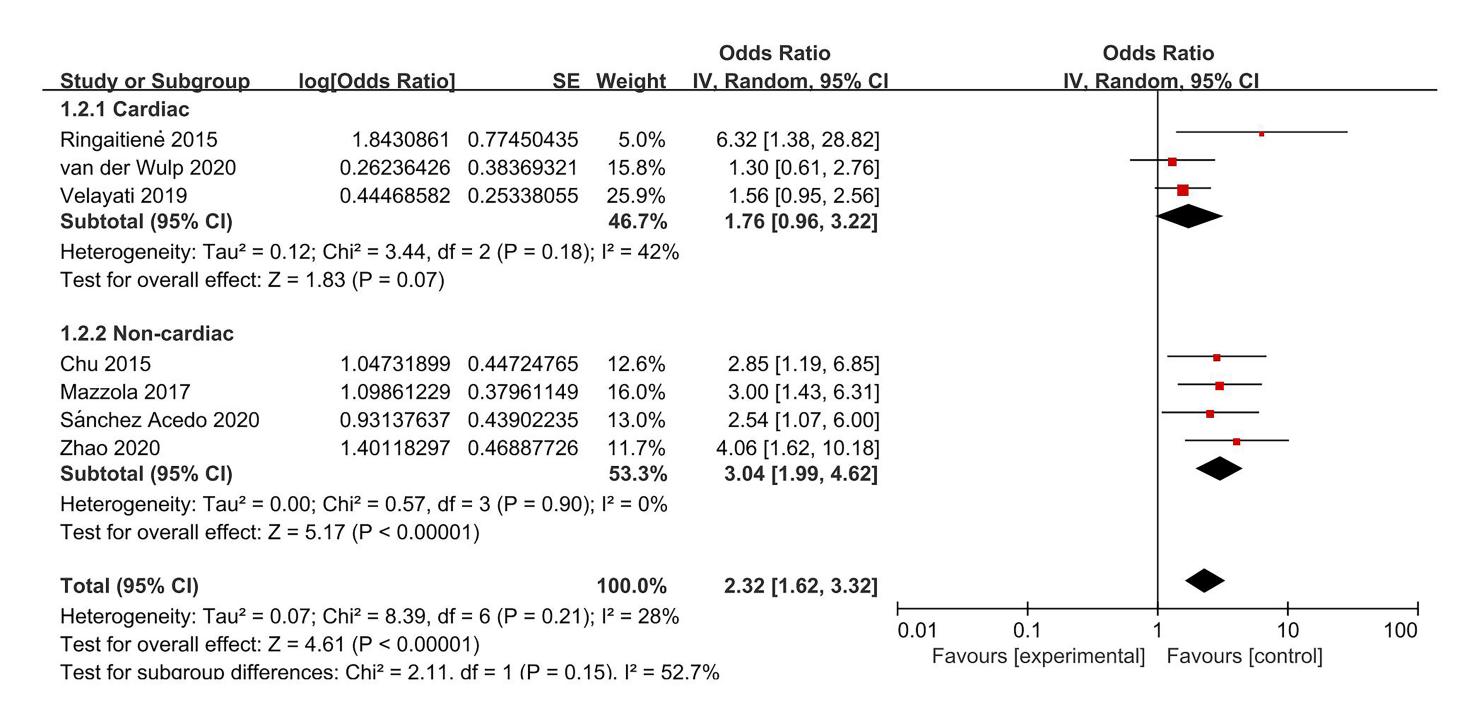


**Supplementary Figure S3.** Forest plot for the subgroup analysis based on methods for evaluating malnutrition.


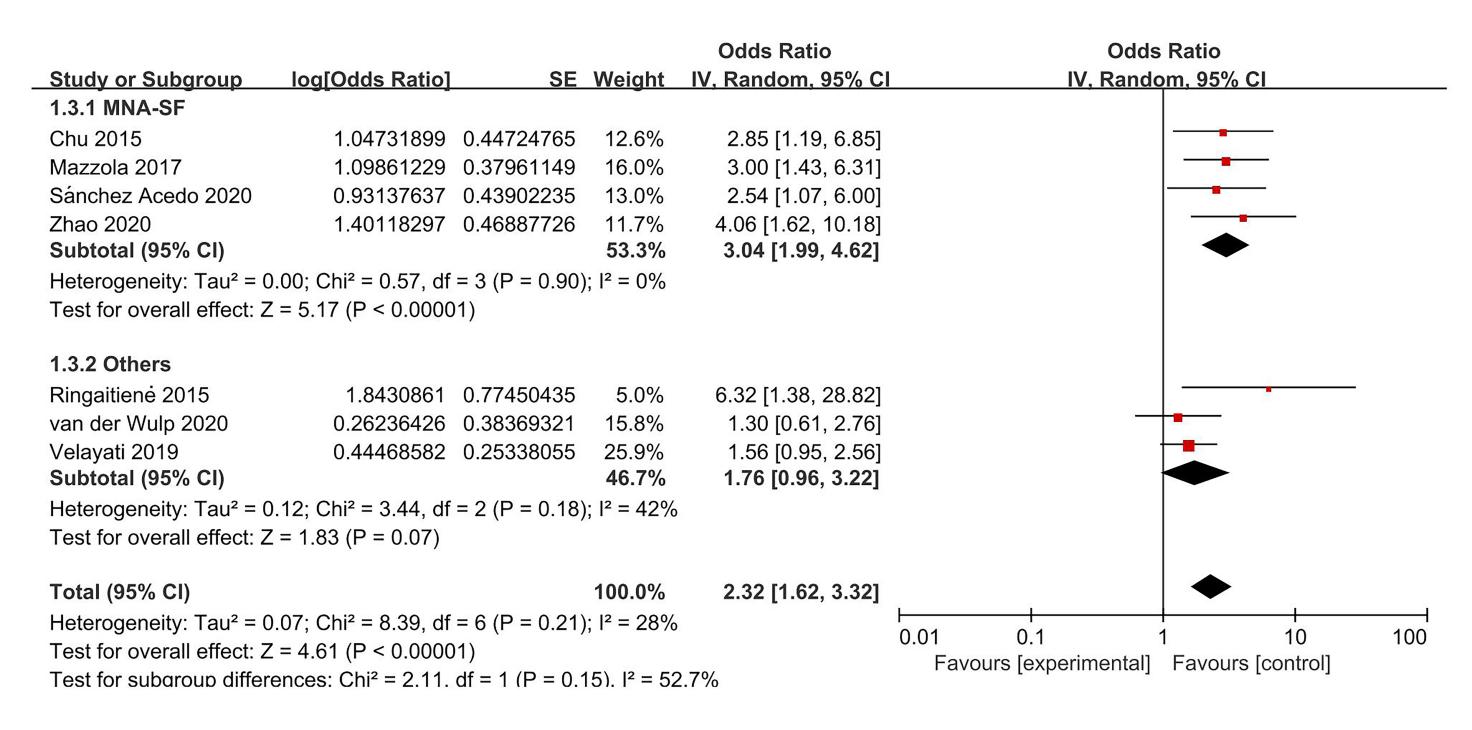


**Supplementary Figure S4.** Forest plot for the subgroup analysis based on methods for evaluating POD.


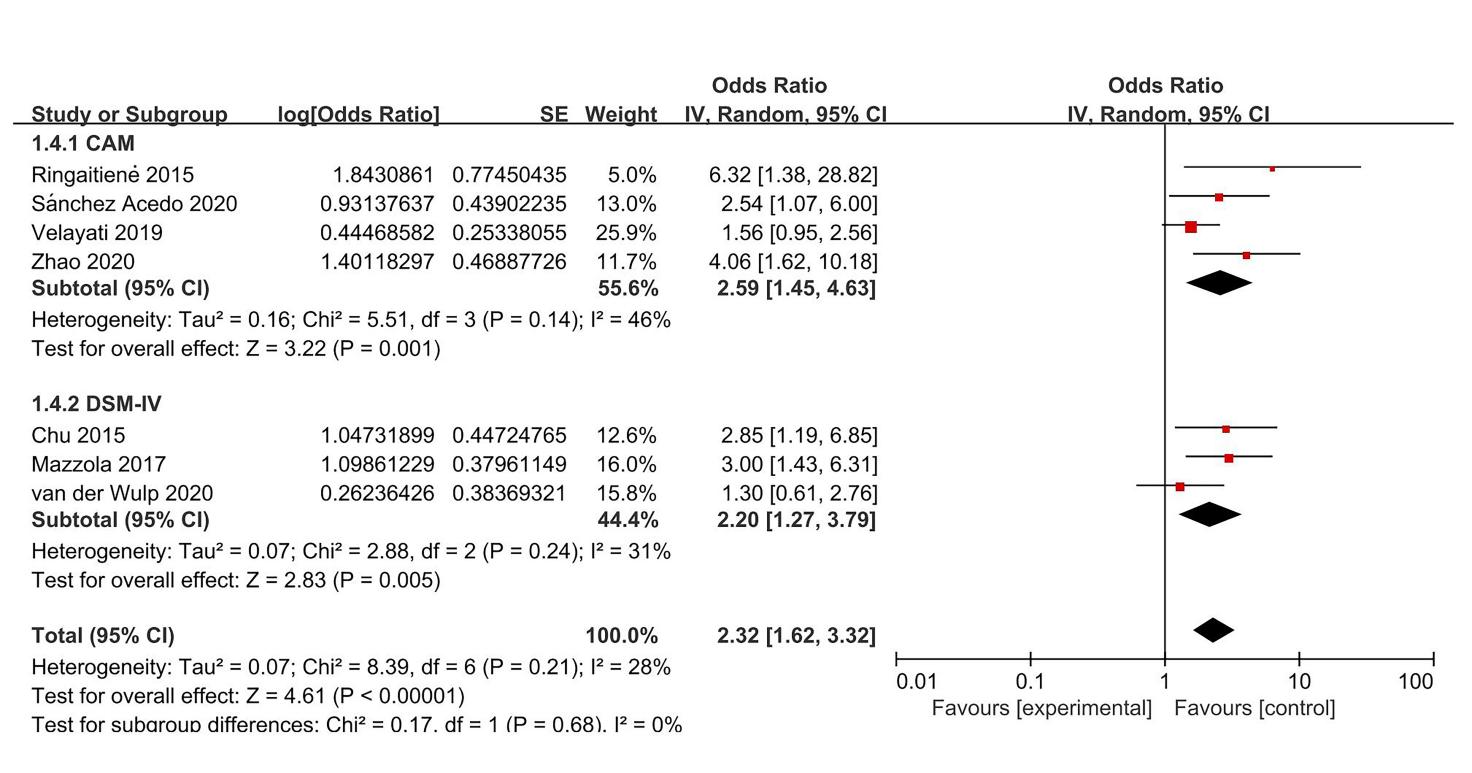


**Supplementary Figure S5.** Forest plot for the subgroup analysis based on quality of the study.


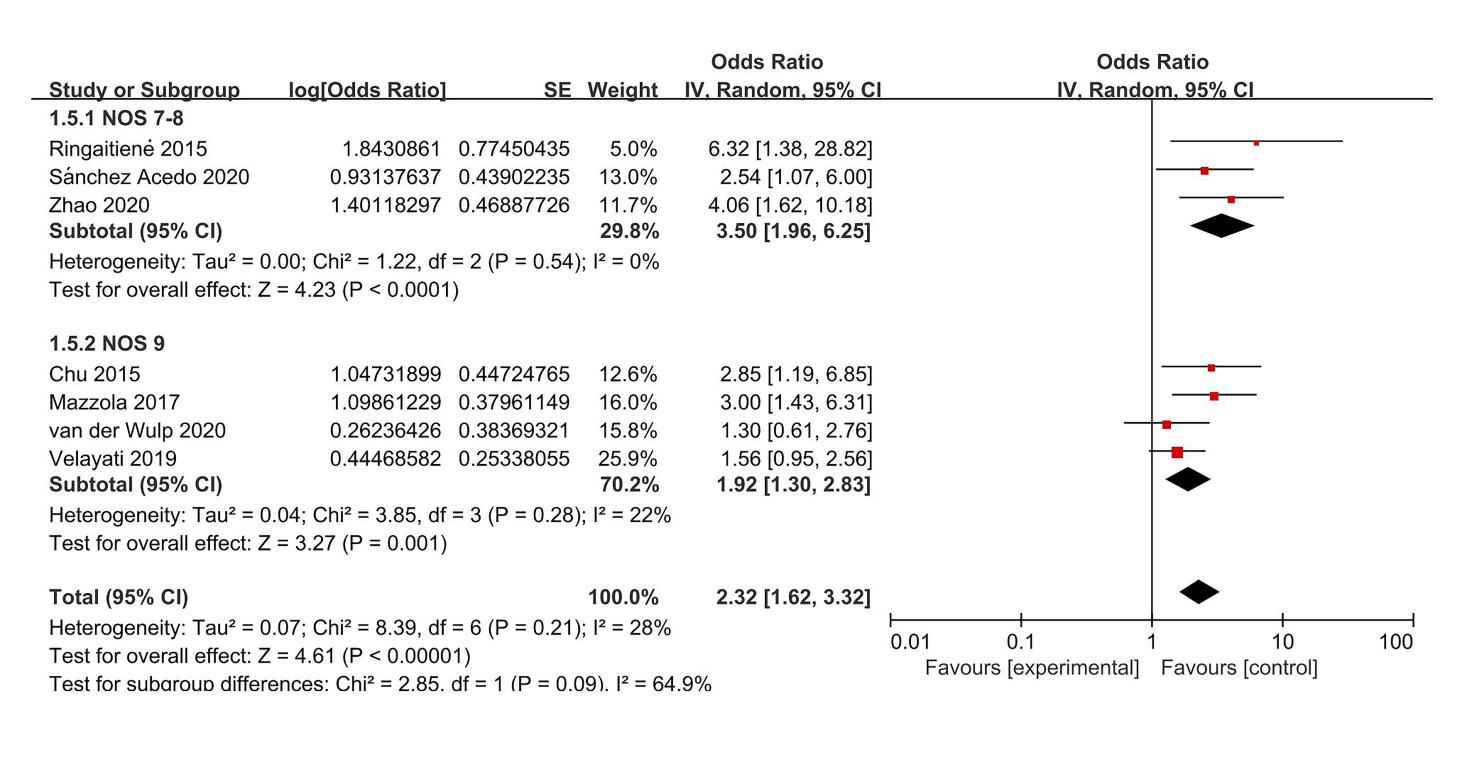

Supplement: Supplementary file 1 — Additional file 1: Supplementary Fig. S1. Forest plot for the subgroup analysis based on location of study. Supplementary Fig. S2. Forest plot for the subgroup analysis based on type of surgery. Supplementary Fig. S3. Forest plot for the subgroup analysis based on methods for evaluating malnutrition. Supplementary Fig. S4. Forest plot for the subgroup analysis based on methods for evaluating POD. Supplementary Fig. S5. Forest plot for the subgroup analysis based on quality of the study. [file 13741_2023_345_MOESM1_ESM.docx]
